# Primary Extradural Meningioma: A Systematic Review of Diagnostic Features, Clinical Management, and Surgical Outcomes

**DOI:** 10.3390/cancers16233915

**Published:** 2024-11-22

**Authors:** Kishore Balasubramanian, Jeffrey A. Zuccato, Abdurrahman F. Kharbat, Christopher Janssen, Nancy M. Gonzalez, Ian F. Dunn

**Affiliations:** 1 Department of Neurosurgery, University of Oklahoma College of Medicine, Oklahoma City, OK 73104, USA; kishore.b@tamu.edu (K.B.); jeffrey.zuccato@ouhealth.com (J.A.Z.); abdurrahman-kharbat@ouhsc.edu (A.F.K.); 2 Division of Neurosurgery, Texas A&M University College of Medicine, Houston, TX 77030, USA; cnjanssen@tamu.edu (C.J.); nancymizeg@tamu.edu (N.M.G.)

**Keywords:** central nervous system tumor, meningioma, systematic review

## Abstract

**Objective:** This systematic review consolidates the literature on primary extradural meningiomas (PEMs), a rare subset of meningiomas. We describe the clinical features, management strategies used, and treatment outcomes for published cases. **Methods:** A systematic review was conducted using PRISMA guidelines across multiple databases to 29 July 2024. Inclusion criteria were adult patients with primarily extradural meningioma and where individual patient clinical data were provided. **Results:** Of 216 studies that met the initial search criteria, 41 satisfied the final inclusion criteria. These 41 studies included 82 patients with 84 total PEMs. The cohort was balanced between sexes with a median age of 46 (range 18–82). Frequent symptoms at initial presentation included pain/headache (46%), weakness (44%), paresthesias (24%), and a palpable superficial mass (23%). The median duration of symptoms to diagnosis was 11 months (range 0.75–120). Surgical resection was the primary treatment approach, achieving a gross total resection in 67% of cases. The majority of lesions were classified as WHO grade 1 (87%). A recurrence was identified during the published follow-up in 11% of cases and a higher WHO grade was expectedly associated with a greater risk of recurrence. The described practice was to use adjuvant radiotherapy in recurrent and high-grade cases. Most cranial lesions were located in the frontal bone, while most spinal lesions affected the cervical spine. Post-treatment symptom improvement or resolution was described in almost all patients at the last follow-up. **Conclusions:** In comparison to intradural meningiomas, PEMs largely follow a more indolent course with a longer duration of symptoms prior to diagnosis, more benign symptoms, a higher proportion of grade 1 tumors, and favorable outcomes; however, there is a small subset of PEMs with extension outside the cranium and spine that require specific considerations for management.

## Simple Summary

This systematic review examines primary extradural meningiomas (PEMs), a rare subset of meningiomas that originate outside the dura mater. This study analyzed 41 publications, including 82 patients with 84 PEMs. PEMs showed a more indolent course compared to intradural meningiomas, with a longer median symptom duration of 11 months before diagnosis. Common presentations included pain/headache, weakness, and palpable masses. Radiographically, PEMs were typically well-defined, bony extra-axial masses. All patients underwent surgical resection, with gross total resection achieved in 67% of cases. The majority (87%) were WHO grade 1 tumors. Recurrence occurred in 11% of cases during follow-up, with a higher WHO grade associated with increased recurrence risk. Adjuvant radiotherapy was used for recurrent and high-grade cases. Most patients showed symptom improvement or resolution at the last follow-up. This review highlights the need for a multidisciplinary approach in managing PEMs and calls for long-term studies to better understand their natural history and outcomes.

## 1. Introduction

Primary extradural meningiomas (PEMs) are a rare subset of meningiomas that arise from arachnoid cells located outside of the dura mater. They most commonly arise and grow in the calvarium, paranasal sinuses and nasal cavity, and vertebrae and may have a component of intradural extension [1,2]. PEMs are rare and account for 0.8–1.8% of all meningiomas, with the majority of meningiomas arising intradurally from arachnoid cap cells [1,2].

PEMs are not well-described in the literature due to rarity, with existing work limited mainly to case reports and small case series. Pathophysiologically, they are postulated to originate from arachnoid cap cells that are displaced to extradural locations during embryonic development due to trauma or during surgical procedures [1,2,3,4]. Available small reviews describe a bimodal age distribution, with the highest incidences in the first and after the fifth decade of life, no major difference between sexes, and presenting symptoms of a palpable mass, headache, and location-specific symptoms [1,2].

Classically, intradural meningiomas are managed surgically and outcomes are generally favorable, with increased risk for recurrence in patients with a higher WHO grade or higher Simpson grade of resection. Recently, new prognostic molecular alterations have been identified and used for prognostication [5,6,7]. Treatment approaches used for PEMs have not been robustly evaluated to determine their degree of alignment with practice for intradural meningiomas. Accordingly, there is a need for a current systematic review of the literature on PEMs to summarize how they present and are managed in order to guide clinical practice for these tumors.

Here, we systematically review the literature on PEM management to better characterize the clinical presentation of these patients, management strategies utilized, and patient outcomes. We provide this as a resource so that existing practice for these rare tumors can be synthesized to inform future practice.

## 2. Methods

### 2.1. The Literature Search

A systematic review was conducted using the Preferred Reporting Items for Systematic Reviews and Meta-Analyses (PRISMA) guidelines. PubMed, EMBASE, Web of Science, and Cochrane were searched from the database inception to 29 July 2024, operating the Boolean full-text search [“Extradural meningioma” OR “primary Extradural meningioma” OR “ectopic meningioma”]. Studies were exported to Rayyan and duplicates were deleted. This study was not submitted to a public registry.

### 2.2. Study Selection

Inclusion and exclusion criteria were defined as follows. Articles were included if they (1) included patients over 18 years of age with a histologically confirmed diagnosis of meningioma and radiologically confirmed extradural location; (2) included individual patient data including details of clinical factors, treatment, outcome, and follow-up; and (3) were written in English. Studies were excluded if they (1) were autopsy reports, animal studies, or studies focusing on imaging characteristics, genetics, or histopathology only; (2) were conference abstracts, literature reviews, meta-analyses, systematic reviews, perspectives, or editorials; (3) lacked adequate clinical data; or (4) were non-English or non-peer-reviewed sources.

Two independent reviewers (C.J. and K.B.) screened all titles and abstracts from the initial systematic search and assessed the full texts of articles that met the inclusion criteria. A third reviewer (A.K.) provided arbitration. Eligible papers were included. References of the included studies were also screened to identify additional pertinent studies.

### 2.3. Data Extraction

One reviewer (K.B.) extracted data from each article, which were confirmed independently by two additional reviewers (C.J. and N.G.). Missing data were not reported by authors or could not be differentiated from other data (i.e., individual data not reported in case series). Extracted data included authors, the year published, sample size, age, gender, presenting symptoms and their duration, comorbidities, physical examination findings, radiological findings, surgical intervention and extent of resection, neuropathological findings including histopathology and immunohistochemistry (IHC), adjuvant therapy received, time to recurrence, time of last follow-up and clinical status at follow-up, and survival.

### 2.4. Data Analysis and Quality Assessment

The primary variables of interest were clinical characteristics, management strategies used, and treatment outcomes for patients with PEMs. For each study, two independent authors (K.B. and N.G.) assessed the level of evidence using the *2011 Oxford Centre For Evidence-Based Medicine* guidelines and the risk of bias by applying the Joanna Briggs Institute checklists for case reports and case series [8,9,10]. Meta-analyses were precluded because all included studies had levels IV–V of evidence and hazard ratios could not be deduced.

### 2.5. Statistical Analysis

SPSS V.25 (IBM Corp, Armonk, NY, USA) and Jamovi (The Jamovi Project, open source) were utilized for all statistical analyses. Continuous variables are summarized as medians with ranges and categorical variables are summarized as frequencies with percentages. Paired sample *t*-tests were used to assess relationships between continuous variables. Chi-squared tests were used to assess relationships between categorical variables. A probability value of 0.05 or less was considered significant.

## 3. Results

### 3.1. Study Inclusion

**Figure 1:**
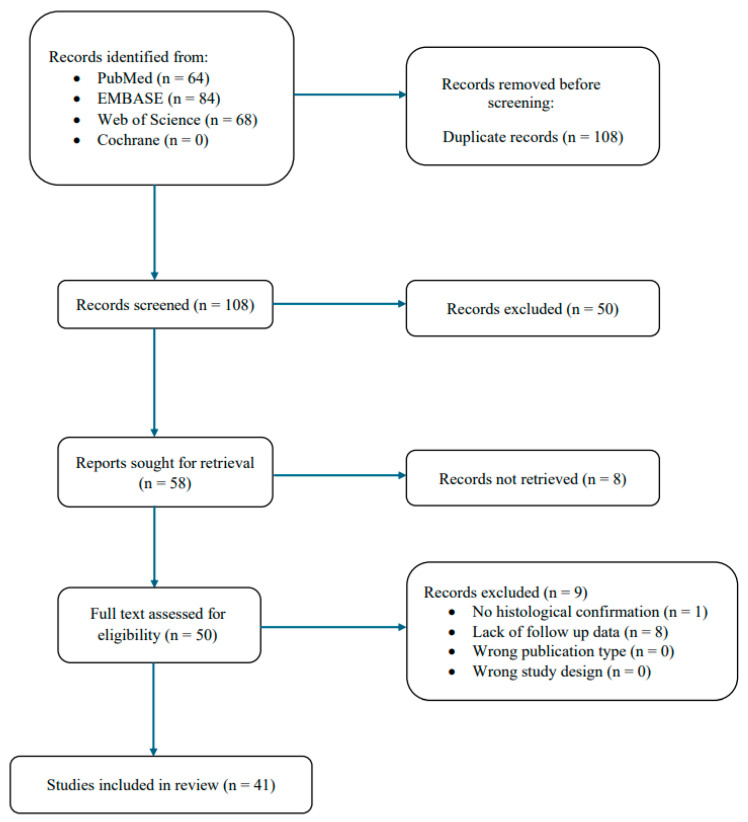
PRISMA 2020 flow diagram.

Figure 1 illustrates the PRISMA flow diagram for the literature search. Our search strategy yielded 216 studies (PubMed: 64, EMBASE: 84, Web of Science: 68, Cochrane: 0), of which 41 met the study inclusion criteria defined a priori. Six were case series and thirty-five were case reports, with IV and V levels of evidence, respectively. All studies and individual patient data for each meningioma patient are outlined in Table 1 [1,2,4,11,12,13,14,15,16,17,18,19,20,21,22,23,24,25,26,27,28,29,30,31,32,33,34,35,36,37,38,39,40,41,42,43,44,45,46,47,48]. Critical appraisal returned a low risk of bias for all included studies (Supplementary Files S1 and S2).

### 3.2. Clinical Presentation

Clinical factors for the study cohort of 82 patients diagnosed with 84 primary extradural meningiomas are shown in Table 2. Both sexes and a spectrum of adult ages were represented in the cohort. The most common presentations were pain/headache (N = 38, 46%), weakness (N = 36, 44%), paresthesias (N = 20, 24%), and a palpable lesion (N = 19, 23%) although a range of additional clinical features may be present depending on tumor location. The median duration of symptoms prior to clinical presentation was 11 months, with a range of 3 weeks to 10 years. Most patients in this cohort did not have major comorbidities described.

### 3.3. Radiographic Features

Imaging features were reported for 56 patients (Table 3), with MRI being the most frequently used imaging modality (N = 50, 89%) followed by CT (N = 23, 41%). There was a balanced distribution of tumors between the cranial (N = 42, 51%) and spinal regions (N = 41, 48%), with one patient having a scapular tumor. Among the cranial lesions, the frontal bone was the most common site (N = 18, 43%) followed by the parietal bone (N = 10, 24%) and temporal bone (N = 7, 17%). For spinal lesions, the cervical region was the most frequently affected (N = 27, 66%) followed by thoracic (N = 18, 44%), and lumbar (N = 1, 2%). Multi-level spinal lesions were more common than single-level lesions, occurring in 63% of patients (N = 25). Invasion and extension into adjacent structures or spaces were assessed in 41 cases and were not identified in 41% (N = 17). Cases with extension included into the intradural compartment (N = 11, 27%); neural structures including cranial, spinal, and peripheral nerves (N = 6, 15%); vasculature structures (N = 3, 7%); and surrounding non-neural tissue (N = 3, 7%).

### 3.4. Clinical Management Approaches and Patient Outcomes

All patients underwent surgical resection. The extent of surgical resection was reported in 81 patients (Table 4). A gross total resection (GTR) was achieved in 67% of cases (N = 54), with a subtotal resection (STR) in 33% (N = 27). Post-operative imaging was conducted in 16 patients, with MRI used in 88% (N = 14) and CT in 12% (N = 2). Post-operative imaging showed no definitive residual tumor in 71% (N = 10), while residual tumor was identified on imaging in 29% of cases (N = 4). Adjuvant radiotherapy was administered to seven patients (37%). The median length of the follow-up was 24 months (range: 3–193 months). Symptom assessment at the last follow-up for 43 patients showed improvement in 58% (N = 25) and resolution in 42% (N = 18). No patients reported a lack of improvement or worsening of symptoms.

### 3.5. Neuropathological Features

WHO grading was available for 54 patients, with 87% (N = 47) being grade 1, 7% (N = 4) grade 2, and 6% of cases (N = 3) grade 3. Among the 68 patients with meningioma histological subtyping data, the most common subtypes were meningothelial (N = 31, 47%) and psammomatous (N = 16, 24%). A total of 8% (N = 5) were atypical and 5% (N = 3) were anaplastic.

IHC staining results were available for 16 patients (Table 5). Epithelial membrane antigen (EMA) was positive in all cases. Negative IHC staining was identified for S100 in five cases (31%), SOX10 in three cases (19%), and CD34 in two cases (13%). The Ki67 index was measured in 25 cases, with 96% (N = 24) showing a Ki67 index of ≤5%.

### 3.6. Evaluation of Potential Clinical Predictors of Recurrence

Recurrence was evaluated in 81 patients, with 11% (N = 9) experiencing recurrence during their observed follow-up (Table 6). WHO grade, tumor location, and extent of resection were assessed for potential prognostic utility in predicting recurrence. A higher WHO grade was correlated with increased recurrence risk, with rates of recurrence of 0–4% in grade 1–2 and 50% in grade 3 (χ^2^ *p* = 0.02). There was a non-significant trend toward higher recurrence in cranial compared to spinal tumors (16.7% versus 5%, χ^2^ *p* = 0.091) as well as with STR compared to GTR (18.5% versus 7.3%, χ^2^ *p* = 0.126). From the full cohort, only two deaths (2%) were reported at the last follow-up and so potential predictors of survival outcomes could not be evaluated.

### 3.7. Characterization by Location

Subgroup analyses were performed according to tumor location as cranial or spinal. There was a statistically significant correlation between the extent of resection and location and a higher proportion of GTRs in cranial than spinal PEMs (85.7% versus 47.4%, χ^2^ *p* < 0.001). There were non-significant trends toward a slightly older age (median 48 versus 43, *t*-test *p* = 0.093) and a longer duration of symptoms (median 12 months versus 9 months, *t*-test *p* = 0.456) in cranial versus spinal PEMs.

## 4. Discussion

This systematic review provides new insights into the clinical presentation, management strategies, and outcomes for primary extradural meningiomas (PEMs), a rare but clinically significant subset of meningiomas that are not well-characterized currently. Although similar to intradural meningiomas in the cell of origin, PEMs are defined by their site of origin being extradural and have unique considerations in how they present and are managed. This study consolidates the existing literature on the clinical experience in managing PEMs from 46 small studies/reports with a total of 86 patients.

In comparison to intradural meningiomas, PEMs showed a more indolent clinical course. The median duration of symptoms prior to diagnosis was 11 months with an upper range limit of 10 years. This is significantly greater than the duration for intradural meningiomas, where half of the patients have a symptom duration of less than 6 months and most have a duration under 2 years [49]. Although both PEMs and intradural meningioma can present with pain/headache and neurological deficits, PEMs are more likely to present with cosmetic changes including a palpable mass or proptosis (observed in a quarter of patients) and did not present with seizure as do many patients with intradural meningioma [49]. Additionally, there was a higher proportion of WHO grade 1 meningiomas in this PEM cohort compared to the literature on intradural meningioma (87% vs. 80%) [50]. Accordingly, outcomes are comparatively good with PEMs, with low rates of recurrence and mortality related to the disease progression in our dataset as well as in others [51,52,53,54].

Radiographically, PEMs are typically well-defined often bony extra-axial masses that may exhibit bony erosion, sclerosis, or hyperostosis [40,55,56]. In contrast, IMs are dural-based tumors that often have a characteristic dural tail and are more likely to have peritumoral edema [57,58]. Although PEMs tend to be more indolent, there is a subset of PEMs that extend into structures outside of the cranium and spine including the brachial plexus (N = 3), paraspinal muscles (N = 1), and thoracic wall (N = 1). This degree of spread is important to evaluate for PEMs as it will impact treatment, the extent of resection, and outcomes. The radiographical differential diagnosis for PEMs also differs from intradural meningiomas given the tumor location, with PEM differentials including primary and metastatic bone tumors [33,59,60,61].

All PEMs in this cohort were managed surgically initially, which differentiates their management from intradural meningiomas that are treated initially with radiotherapy in one-quarter of cases [49]. This is likely due to the atypical imaging findings of these rare meningiomas that prompts surgical resection to obtain a neuropathological diagnosis, which may not be required for intradural meningiomas with classic imaging features that are amenable to treatment with radiation.

The extent of resection of PEMs is highly impacted by tumor location and the presence of invasion into surrounding structures. In this cohort, the overall gross total resection (GTR) rate was 68%, and there was a higher proportion of GTRs in cranial (86%) than spinal (47%) cases. In comparison to PEMs, IMs tend to lower GTR proportion in cranial lesions (45–79%) and a higher GTR proportion in spinal tumors (up to 94%) [62,63,64,65,66]. These differences are likely attributed to intradural meningioma invasion into surrounding parenchyma in cranial lesions and PEMs extending into extraspinal structures like the brachial plexus and paraspinal muscles in spinal meningiomas. The goal for both PEMs and intradural meningioma is to maximize the extent of resection as is feasible according to the Simpson grade to reduce the risk of recurrence [67].

Molecular alterations in meningioma have been well-characterized, including characteristic mutations such as NF2, copy number alterations, and DNA methylation signatures and integrated molecular classifications of meningioma have been proposed [5,6,7,68,69]. It will be important for future work to evaluate molecular alterations specific to PEMs to improve our understanding of these tumors and guide treatment. Unfortunately, this is beyond the scope of this review due to the limited published data available.

There are similarities in how PEMs and intradural meningioma present and are managed that are important to note. The median age in this cohort of 46 years aligns with what has been shown for intradural meningiomas that primarily present in the fifth decade of life [70]. We show a slight female predominance in PEMs (60%), while intradural meningiomas have a much higher female predominance with a 3–4:1 in grade 1 tumors [71,72]. Clinical practice for PEMs in this cohort was to manage surgically upfront and to treat with radiation therapy in higher-grade tumors or recurrent disease, and these same subsets of patients receive radiotherapy in intradural meningioma [73]. PEM subgroups with trends toward increased recurrence were those with a higher WHO grade and subtotal resection, similar to recurrence predictors in intradural meningioma.

### Limitations

This review is limited to the individual patient data in the literature, consisting mainly of a small number of case reports and small case series. The rarity of these tumors precluded larger, more robust studies and led to the need for a comprehensive review of clinical practice for these patients. Additionally, there was significant heterogeneity in reporting across the included studies, leading to the incomplete data for some variables limiting our ability to perform a meta-analysis. The nature of this study, with its reliance on case reports and case series, predisposes this study to publication bias. Long-term follow-up in larger cohorts with standardized reporting will be required for future studies and meta-analyses to further characterize the clinical course and optimal management of PEMs, especially as it relates to the emerging importance of molecular features.

## 5. Conclusions

Primary extradural meningiomas (PEMs) represent a rare but clinically significant subset of meningiomas. Although they present with symptoms similar to other extradural masses depending on location, PEMs are meningiomas and are expected to grow and respond to therapy as intradural meningiomas, and outcomes are generally favorable. Optimal management requires a multidisciplinary approach involving radiologists, neurosurgeons, and pathologists, with surgical resection remaining the standard-of-care front-line treatment for symptomatic or growing lesions. Long-term follow-up studies are needed to build our understanding of the natural history and long-term outcomes of PEMs.

## Figures and Tables

**Table 1:** Description of included individual patient data.

Study #	Author	Year	Age	Sex	Cranial vs. Spinal	Location	Extent of Resection	WHO Grade	Histopathological Subtype	Recurrence	Current Status
1	Echalier et al. [11]	2024	25	M	Spinal	Cervical	Total	1	Mixed	No	A
2	Crene et al. [4]	2024	42	F	Cranial	Frontal bone	Total	1	N/A	No	A
3	Redhu et al. [13]	2024	45	F	Spinal	Cervical-Thoracic	Total	1	N/A	No	A
		2024	50	F	Spinal	Thoracic	Subtotal	N/A	Mixed	Yes	A
4	Hsieh et al. [14]	2023	64	F	Spinal	Cervical	Subtotal	2	Atypical	N/A	A
5	Vijayan et al. [15]	2023	26	F	Spinal	Cervical	Subtotal	1	Transitional	No	A
6	Maiorano et al. [16]	2023	36	F	Cranial	Clivus	Total	N/A	N/A	No	A
7	Almatrafi et al. [48]	2023	24	F	Spinal	Thoracic	Total	1	Psammomatous	No	A
8	Punia et al. [17]	2021	35	M	Cranial	Frontoparietal bone	Subtotal	1	Meningothelial	No	A
9	Nguyen et al. [18]	2021	22	F	Spinal	Cervical	Total	N/A	Psammomatous	No	A
10	Shui et al. [19]	2021	66	F	Spinal	Thoracic	Subtotal	1	Meningothelial	No	A
11	Zhan et al. [20]	2019	47	F	Spinal	Cervical	Subtotal	1	Meningothelial	No	A
12	Slentz et al. [21]	2018	76	F	Cranial	Orbit	Total	3	Malignant	No	D
13	Lai et al. [22]	2018	35	M	Spinal	Cervical	Subtotal	1	Meningothelial	No	A
14	Mankotia et al. [37]	2018	27	F	Cranial	Temporal bone	Total	1	N/A	No	A
15	Ghanchi et al. [23]	2018	40	M	Spinal x2	Thoracic and Lumbar	Total	1	N/A	No	A
16	Sivaraju et al. [24]	2017	50	M	Spinal	Cervical	Subtotal	1	Psammomatous	No	A
17	Pant et al. [25]	2017	50	M	Spinal	Cervical	Subtotal	1	Meningothelial	No	A
18	Ito et al. [26]	2017	41	F	Spinal	Thoracic	Total	N/A	Psammomatous	No	A
19	Hong et al. [27]	2017	58	F	Spinal	Thoracic	Total	2	Atypical	No	A
20	Dehcordi et al. [28]	2016	39	F	Spinal x2	Thoracic x2	Total	N/A	Meningothelial	No	A
21	Pandey et al. [38]	2016	18	M	Spinal	Thoracic	Total	N/A	Psammomatous	No	A
22	Bettaswamy et al. [29]	2016	50	M	Spinal	Cervical	Subtotal	1	Meningothelial	No	A
		2016	41	M	Spinal	Cervical	Total	1	Meningothelial	No	A
23	Wu et al. [30]	2014	62	F	Spinal	Thoracic	Total	1	Psammomatous	No	A
		2014	42	M	Spinal	Cervical	Subtotal	1	Psammomatous	No	A
		2014	40	F	Spinal	Cervical-Thoracic	Subtotal	1	Meningothelial	No	A
		2014	50	M	Spinal	Cervical	Subtotal	1	Fibroblastic	No	A
		2014	27	F	Spinal	Cervical	Total	1	Psammomatous	No	A
		2014	29	M	Spinal	Cervical	Subtotal	1	Psammomatous	No	A
		2014	32	F	Spinal	Cervical	Subtotal	1	Meningothelial	No	A
		2014	39	F	Spinal	Thoracic	Total	1	Psammomatous	No	A
		2014	45	M	Spinal	Cervical	Subtotal	1	Meningothelial	Yes	A
		2014	41	M	Spinal	Cervical	Total	1	Transitional	No	A
		2014	28	F	Spinal	Cervical	Subtotal	1	Meningothelial	No	A
		2014	44	F	Spinal	Cervical	Subtotal	1	Psammomatous	No	A
24	Kariyattil et al. [39]	2014	40	F	Cranial	Frontal bone	Total	1	N/A	No	A
25	Pushker et al. [31]	2013	30	F	Cranial	Orbit	Subtotal	N/A	N/A	Yes	A
		2013	40	M	Cranial	Orbit	Subtotal	N/A	Meningothelial	Yes	A
26	Mattox et al. [40]	2011	61	M	Cranial	Parietal bone	Total	1	Atypical	No	A
27	Uygur et al. [47]	2010	63	F	Cranial	Sphenoid bone	Total	1	Meningothelial	No	A
28	Liu et al. [2]	2010	38	M	Cranial	Temporal bone	Total	1	Meningothelial	No	A
		2010	26	M	Cranial	Frontotemporal bone	Total	1	Psammomatous	No	A
		2010	26	M	Cranial	Parietal bone	Total	1	Meningothelial	No	A
		2010	41	M	Cranial	Parietal bone	Total	1	Psammomatous	No	A
		2010	53	F	Cranial	Temporal bone	Total	1	Meningothelial	No	A
29	Benzagmout et al. [32]	2009	65	F	Spinal	Cervical-Thoracic	Subtotal	1	Meningothelial	No	A
30	Frank et al. [33]	2008	45	F	Spinal	Cervical	Subtotal	N/A	Psammomatous	No	A
31	Llauger et al. [34]	2007	82	F	Ex	Scapula	Total	N/A	N/A	No	A
32	Bassiouni et al. [12]	2006	47	M	Cranial	Parietal bone	Total	N/A	Regressive	No	A
		2006	60	M	Cranial	Frontal bone	Total	N/A	Meningothelial	No	A
		2006	31	M	Cranial	Frontal bone	Total	N/A	Fibroblastic	No	A
		2006	35	F	Cranial	Frontal bone	Total	N/A	Meningothelial	No	A
		2006	62	M	Cranial	Frontal bone	Total	N/A	Meningothelial	No	A
		2006	46	F	Cranial	Parietal bone	Total	N/A	Meningothelial	Yes	A
		2006	60	F	Cranial	Frontal bone	Total	N/A	Fibroblastic	No	A
		2006	57	F	Cranial	Parietal bone	Total	N/A	Fibroblastic	No	A
		2006	72	F	Cranial	Frontal bone	Total	N/A	Transitional	No	A
		2006	63	F	Cranial	Frontal bone	Total	N/A	Meningothelial	No	A
		2006	62	F	Cranial	Frontal bone	Total	N/A	Atypical	No	A
		2006	54	F	Cranial	Frontal bone	Total	N/A	Meningothelial	No	A
		2006	34	M	Cranial	Temporal bone	Total	N/A	Meningothelial	No	A
		2006	52	M	Cranial	Parietal bone	Total	N/A	Transitional	Yes	A
		2006	70	F	Cranial	Parietal bone	Total	N/A	Malignant	Yes	A
		2006	80	F	Cranial	Frontal bone	Total	N/A	Meningothelial	No	A
33	Takeuchi et al. [35]	2005	50	M	Spinal	Cervical	Subtotal	N/A	Meningothelial	No	A
34	Tokgoz et al. [41]	2005	44	M	Cranial	Frontoparietal bone	Total	2	Chordoid	No	A
35	Restrepo et al. [36]	2005	57	F	Spinal	Cervical-Thoracic	Total	1	Psammomatous	No	A
36	Zevgaridis et al. [42]	2002	75	F	Spinal	Thoracic	Total	1	Psammomatous	No	A
37	Yamazaki et al. [43]	2001	62	M	Cranial	Posterior fossa	Total	1	Meningothelial	No	A
38	Buchfelder et al. [44]	2001	72	F	Spinal	Cervical-Thoracic	Total	1	Meningothelial	No	A
39	Lang et al. [1]	2000	41	F	Cranial	Sphenoid bone	Total	1	N/A	No	A
		2000	49	F	Cranial	Frontal bone	Total	1	N/A	No	A
		2000	68	M	Cranial	Frontal bone	Total	1	N/A	No	A
		2000	47	F	Cranial	Nasal cavity	Total	1	N/A	Yes	A
		2000	50	F	Cranial	Nasal cavity	Subtotal	3	Malignant	Yes	D
		2000	67	F	Cranial	Temporal bone	Total	1	N/A	No	A
		2000	59	M	Cranial	Sphenoid bone	Subtotal	2	Atypical	No	A
		2000	18	M	Cranial	Petrous bone	Subtotal	1	N/A	No	A
40	Qasho et al. [45]	1998	46	F	Cranial	Frontal bone	Total	1	Fibroblastic	No	A
41	Salvati et al. [46]	1991	20	M	Spinal	Thoracic	N/A	N/A	Transitional	No	A

F, female; M, male; A, alive; D, deceased; N/A, not available.

**Table 2:** Clinical presentation.

Characteristics (N = 82)	N or Median	% or Range
Demographics		
Gender (male)	33	40
Age	46	18–82
Presenting signs and symptoms *		
Pain/headache	38	46
Weakness	36	44
Paresthesias	20	24
Palpable mass	19	23
Urinary incontinence	10	12
Headache	9	11
Gait disturbance	7	9
Positive Babinski sign	6	7
Proptosis	4	5
Visual changes	3	4
Abnormal reflexes	2	2
Nausea	2	2
Auditory changes	1	1
Muscle atrophy	1	1
Duration of symptoms (months)	11	0.75–120
Comorbidities		
Trauma	2	25
HIV	1	13
Seizures **	1	13
Frozen shoulder	1	13
Metabolic disease	1	13
History of metastatic disease	1	13

* Some patients may fit multiple categories. Data represent the frequency of the individual findings in relation to the total sample size. ** History of seizure disorder/seizures not thought to be caused by meningioma.

**Table 3:** Summary of clinical workup.

Clinical Summary	N	%
Radiological Workup (N = 56) *		
MRI	50	89
CT	23	41
Ultrasound	6	11
X-ray	1	2
Tumor Location (N = 84)		
Cranial	42	51
Spinal	41	48
Scapula	1	1
Cranial Lesions (N = 42) *		
Frontal bone	18	43
Parietal bone	10	24
Temporal bone	7	17
Orbit	3	7
Sphenoid bone	3	7
Nasal cavity	2	5
Clivus	1	2
Posterior fossa	1	2
Spinal Lesions (N = 41) *		
Cervical	27	66
Thoracic	18	44
Lumbar	1	2
Extent of Spinal Lesions (N = 41)		
Multi-level	25	63
Single level	16	37
Involvement of adjacent structures (N = 41)		
None	17	41
Intradural extension	11	27
Neural structures	6	15
Vascular structures	3	7
Surrounding non-neural tissue	3	7

* Some patients may fit multiple categories. Data represent the frequency of modality in relation to the total sample size.

**Table 4:** Summary of clinical management and patient outcomes.

Clinical Management	N or Median	% or Range
Extent of resection (N = 81)		
Gross total	54	67
Subtotal	27	33
Post-operative imaging modality used (N = 16)		
MRI	14	88
CT	2	12
Post-operative imaging findings (N = 14)		
No definitive residual tumor	10	71
Residual tumor present	4	29
Adjuvant therapy received (N = 19)		
No	12	63
Yes	7	37
Length to last follow-up (N = 56, months)	24	3–193
Symptom status at last follow-up (N = 43)		
Improved	25	58
Resolved	18	42
No improvement	0	0
Worsened	0	0
WHO grade (N = 54)		
1	47	87
2	4	7
3	3	6
Histological subtype (N = 66) *		
Meningothelial	31	47
Psammomatous	16	24
Atypical	5	8
Transitional	5	8
Fibroblastic	5	8
Anaplastic	3	5
Metaplastic	1	2
Chordoid	1	2
Recurrence status at last follow-up (N = 81)		
No	72	89
Yes	9	11
Survival status at last follow-up (N = 82)		
Alive	80	98
Deceased	2	2

* Assessment of histological subtype was limited to the details provided in the source manuscripts.

**Table 5:** Summary of IHC staining results.

	N	%
Positive IHC staining (N = 16) *		
Epithelial membrane antigen (EMA)	16	100
Vimentin	7	43
Progesterone	3	19
Cytokeratin (CK)	1	6
Somatostatin receptor 2 (SSTR2)	1	6
Negative IHC staining (N = 16) *		
S100	5	31
SOX10	3	19
CD34	2	13
CD99	1	6
CK	1	6
Ki67 Index (N = 25)		
≤5%	24	96
>5%	1	4

* Some patients may have multiple positive/negative IHC stains. Data represent the frequency of the individual findings in relation to the total sample size.

**Table 6:** Summary of patients with recurrence.

Author	Age	Cranial vs. Spinal	Location	Extent of Resection	WHO Grade	Histopathological Subtype	Disease-Free Survival (Months)	Follow-Up Intervention	Last Follow-Up (Months)	Status at Last Follow-Up	Current Status
Redhu et al. [13]	50	Spinal	Thoracic	Subtotal	N/A	Mixed	48	Surgery	54	NED	A
Wu et al. [30]	45	Spinal	Cervical	Subtotal	1	Meningothelial	88	Surgery	168	NED	A
Pushker et al. [31]	30	Cranial	Orbit	Subtotal	N/A	N/A	8	Surgery	26	NED	A
Pushker et al. [31]	40	Cranial	Orbit	Subtotal	N/A	Meningothelial	11	Surgery	24	NED	A
Bassiouni et al. [12]	46	Cranial	Parietal bone	Total	N/A	Meningothelial	36	N/A	N/A	N/A	A
Bassiouni et al. [12]	70	Cranial	Parietal bone	Total	N/A	Malignant	8	N/A	N/A	N/A	A
Bassiouni et al. [12]	52	Cranial	Parietal bone	Total	N/A	Transitional	30	N/A	N/A	N/A	A
Lang et al. [1]	47	Cranial	Nasal cavity	Total	1	N/A	126	Surgery	193	NED	A
Lang et al. [1]	50	Cranial	Nasal cavity	Subtotal	3	Malignant	120	Surgery	136	Metastasis	D

N/A, not available; NED, no evidence of disease; A, alive; D, deceased.

## Supplementary Materials

The following supporting information can be downloaded at: https://www.mdpi.com/article/10.3390/cancers16233915/s1, File S1: Joanna Briggs Institute Checklist for Case Series; File S2: Joanna Briggs Institute Checklist for Case Reports.
