# Supplementary material for: Primary Extradural Meningioma: A Systematic Review of Diagnostic Features, Clinical Management, and Surgical Outcomes
Source: Cancers (Basel). 2024 Nov 22;16(23):3915. doi: 10.3390/cancers16233915 (PMC11640065; doi:10.3390/cancers16233915)
Supplement: Supplementary file 1 [file cancers-16-03915-s001.zip › Supplementary File S1.pdf]

S1: Joanna Briggs Institute Checklist for Case Series

Joanna Briggs Institute Checklist for Case Series												
**Study author**	**Year**	**1**	**2**	**3**	**4**	**5**	**6**	**7**	**8**	**9**	**10**	**Rating**
Redhu et al.	2024	Yes	Yes	Yes	No	Yes	Yes	Yes	Yes	No	NA	Good
Wu et al.	2014	Yes	Yes	Yes	No	Yes	Yes	Yes	Yes	No	NA	Good
Pushker et al.	2013	Yes	Yes	Yes	No	Yes	Yes	Yes	Yes	No	NA	Good
Bassiouni et al.	2006	Yes	Yes	Yes	No	Yes	Yes	Yes	Yes	No	NA	Good
Liu et al.	2010	Yes	Yes	Yes	No	Yes	Yes	Yes	Yes	No	NA	Good
Bettaswamy et al.	2016	Yes	Yes	Yes	No	Yes	Yes	Yes	Yes	No	NA	Good
Lang et al.	2000	Yes	Yes	Yes	No	Yes	Yes	Yes	Yes	No	NA	Good

**Joanna Briggs Institute Checklist for Case Series – Criteria**
1. Were there clear criteria for inclusion in the case series?
2. Was the condition measured in a standard, reliable way for all participants included in the case series?
3. Were valid methods used for identification of the condition for all participants included in the case series?
4. Did the case series have consecutive inclusion of participants?
5. Did the case series have complete inclusion of participants?
6. Was there clear reporting of the demographics of the participants in the study?
7. Was there clear reporting of clinical information of the participants?
8. Were the outcomes or follow up results of cases clearly reported?
9. Was there clear reporting of the presenting site(s)/clinic(s) demographic information?
10. Was statistical analysis appropriate?
**Responses Options: Yes, No, Unclear, Not Applicable (NA)**
**Quality Rating: Poor 0 – 3; Fair 4 – 7; Good 8 – 10**
