# Supplementary material for: Primary Extradural Meningioma: A Systematic Review of Diagnostic Features, Clinical Management, and Surgical Outcomes
Source: Cancers (Basel). 2024 Nov 22;16(23):3915. doi: 10.3390/cancers16233915 (PMC11640065; doi:10.3390/cancers16233915)
Supplement: Supplementary file 1 [file cancers-16-03915-s001.zip › Supplementary File S2.pdf]

S2: Joanna Briggs Institute Checklist for Case Reports

Joanna Briggs Institute Checklist for Case Reports									
**Author**	**Year**	**1**	**2**	**3**	**4**	**5**	**6**	**7**	**8**
Echalier et al.	2024	Yes	Yes	Yes	Yes	Yes	Yes	Yes	Yes
Crene et al.	2024	Yes	Yes	Yes	Yes	Yes	Yes	Yes	Yes
Redhu et al.	2024	Yes	Yes	Yes	Yes	Yes	Yes	Yes	Yes
Hsieh et al.	2023	Yes	Yes	Yes	Yes	Yes	Yes	Yes	Yes
Vijayan et al.	2023	Yes	Yes	Yes	Yes	Yes	Yes	Yes	No
Maiorano et al.	2023	Yes	Yes	Yes	Yes	Yes	Yes	Yes	Yes
Almatrafi et al.	2023	Yes	Yes	Yes	Yes	Yes	Yes	Yes	Yes
Gotecha et al.	2021	Yes	Yes	Yes	Yes	Yes	Yes	Yes	Yes
Nguyen et al.	2021	Yes	Yes	Yes	Yes	Yes	Yes	Yes	Yes
Shui et al.	2021	Yes	Yes	Yes	Yes	Yes	Yes	Yes	No
Zhan et al.	2019	Yes	Yes	Yes	Yes	Yes	Yes	Yes	Yes
Slentz et al.	2018	Yes	Yes	Yes	Yes	Yes	Yes	Yes	Yes
Lai et al.	2018	Yes	Yes	Yes	Yes	Yes	Yes	Yes	No
Manikota et al.	2018	Yes	Yes	Yes	Yes	Yes	Yes	Yes	Yes
Ghanch et al.i	2018	Yes	Yes	Yes	Yes	Yes	Yes	Yes	Yes
Sivaraju et al.	2017	Yes	Yes	Yes	Yes	Yes	Yes	Yes	Yes
Pant et al.	2017	Yes	Yes	Yes	Yes	Yes	Yes	Yes	No
Ito et al.	2017	Yes	Yes	Yes	Yes	Yes	Yes	Yes	Yes
Hong et al.	2017	Yes	Yes	Yes	Yes	Yes	Yes	Yes	Yes
Dehcordi et al.	2017	Yes	Yes	Yes	Yes	Yes	Yes	Yes	Yes
Pandey et al.	2016	Yes	Yes	Yes	Yes	Yes	Yes	Yes	No
Bettaswamy et al.	2016	Yes	Yes	Yes	Yes	Yes	Yes	Yes	Yes
Wu et al.	2014	Yes	Yes	Yes	Yes	Yes	Yes	Yes	Yes
Kiribati et al.	2014	Yes	Yes	Yes	Yes	Yes	Yes	Yes	Yes
Pushker et al.	2013	Yes	Yes	Yes	Yes	Yes	Yes	Yes	No
Austin et al.	2011	Yes	Yes	Yes	Yes	Yes	Yes	Yes	Yes
Uygur et al.	2010	Yes	Yes	Yes	Yes	Yes	Yes	Yes	Yes
Liu et al.	2010	Yes	Yes	Yes	Yes	Yes	Yes	Yes	Yes
Benzagmout et al.	2009	Yes	Yes	Yes	Yes	Yes	Yes	Yes	Yes
Frank et al.	2008	Yes	Yes	Yes	Yes	Yes	Yes	Yes	Yes
Llauger et al.	2007	Yes	Yes	Yes	Yes	Yes	Yes	Yes	Yes
Bassiouni et al.	2006	Yes	Yes	Yes	Yes	Yes	Yes	Yes	Yes
Takeuchi et al.	2005	Yes	Yes	Yes	Yes	Yes	Yes	Yes	No
Tokgoz et al.	2005	Yes	Yes	Yes	Yes	Yes	Yes	Yes	Yes
Restrepo et al.	2005	Yes	Yes	Yes	Yes	Yes	Yes	Yes	Yes
Zevgaridis	2002	Yes	Yes	Yes	Yes	Yes	Yes	Yes	Yes
Yamazaki et al.	2001	Yes	Yes	Yes	Yes	Yes	Yes	Yes	Yes
Buchfelder et al.	2001	Yes	Yes	Yes	Yes	Yes	Yes	Yes	No
Lang et al.	2000	Yes	Yes	Yes	Yes	Yes	Yes	Yes	Yes

**Joanna Briggs Institute Checklist for Case Studies– Criteria**
1. Were patient's demographic characteristics clearly described?
2. Was the patient's history clearly described and presented as a timeline?
3. Was the current clinical condition of the patient on presentation clearly described?
4. Were diagnostic tests or assessment methods and the results clearly described?
5. Was the intervention(s) or treatment procedure(s) clearly described?
6. Was the post-intervention clinical condition clearly described?
7. Were adverse events (harms) or unanticipated events identified and described?
8. Does the case report provide takeaway lessons?
**Responses Options: Yes, No, Unclear, Not Applicable (NA)**
